# Comparison of the Fc glycosylation of fetal and maternal immunoglobulin G

**DOI:** 10.1007/s10719-012-9381-6

**Published:** 2012-05-10

**Authors:** Helga K. Einarsdottir, Maurice H. J. Selman, Rick Kapur, Sicco Scherjon, Carolien A. M. Koeleman, André M. Deelder, C. Ellen van der Schoot, Gestur Vidarsson, Manfred Wuhrer

**Affiliations:** 1 Department of Experimental Immunohematology, Sanquin Research, and Landsteiner Laboratory, Academic Medical Center, University of Amsterdam, Amsterdam, The Netherlands; 2 Department of Immunohematology and Blood Transfusion, Leiden University Medical Center, Leiden, The Netherlands; 3 Department of Parasitology, Biomolecular Mass Spectrometry Unit, Leiden University Medical Center, Albinusdreef 2, 2333 ZA Leiden, The Netherlands

**Keywords:** Fc receptor, Galactosylation, Glycopeptide, Placenta, Sialylation

## Abstract

Human immunoglobulin G (IgG) molecules are composed of two Fab portions and one Fc portion. The glycans attached to the Fc portions of IgG are known to modulate its biological activity as they influence interaction with both complement and various cellular Fc receptors. IgG glycosylation changes significantly with pregnancy, showing a vast increase in galactosylation and sialylation and a concomitant decrease in the incidence of bisecting GlcNAc. Maternal IgGs are actively transported to the fetus by the neonatal Fc receptor (FcRn) expressed in syncytiotrophoblasts in the placenta, providing the fetus and newborn with immunological protection. Two earlier reports described significant differences in total glycosylation between fetal and maternal IgG, suggesting a possible glycosylation-selective transport *via* the placenta. These results might suggest an alternative maternal transport pathway, since FcRn binding to IgG does not depend on Fc-glycosylation. These early studies were performed by releasing N-glycans from total IgG. Here, we chose for an alternative approach analyzing IgG Fc glycosylation at the glycopeptide level in an Fc-specific manner, providing glycosylation profiles for IgG1 and IgG4 as well as combined Fc glycosylation profiles of IgG2 and 3. The analysis of ten pairs of fetal and maternal IgG samples revealed largely comparable Fc glycosylation for all the analyzed subclasses. Average levels of galactosylation, sialylation, bisecting GlcNAc and fucosylation were very similar for the fetal and maternal IgGs. Our data suggest that the placental IgG transport is not Fc glycosylation selective.

## Introduction

Human immunoglobulin G (IgG) represents the most abundant class of immunoglobulins in the circulation with typical concentrations of approximately 10 mg/ml [1]. These B-cell derived immunoglobulins are soluble forms of the B cell receptor, formed after encounter between antigen-specific B cells and its cognate antigen, antigen processing and activation through helper T cells, leading to affinity maturation and class switching to one of the γ-encoding genes of the heavy-chain locus, forming IgG [1, 2]. IgGs occur in different subclasses (IgG1, IgG2, IgG3, and IgG4), named in order of decreasing abundance [1]. They are formed from two heavy chains and two lights chains which together form two fragment antigen binding (Fab) portions and one fragment crystallizable (Fc) portion, which is distinct between the subclasses, and influences their specific function through specific interaction with complement, Fc-gamma receptors (FcγR) and FcRn [3, 4]. The Fab portions contain the (hyper-)variable parts of the molecule which define its binding properties to antigens including pathogen molecular patterns, and are therefore unique for each clonally derived antibody [2]. The Fc portions of all four subclasses of IgG are known to be glycosylated at asparagine 297 [5].

The Asn297-linked glycans of IgG are biantennary complex-type structures which are predominantly core-fucosylated and are in part modified by a bisecting *N*-acetylglucosamine (GlcNAc) [5–8]. Antennae are partially truncated varying in their degree of galactosylation and may carry a sialic acid residue. The Fc glycans of IgG are involved in the interaction with all FcγR besides the neonatal Fc receptor (FcRn) [9, 10]. In addition to direct interaction of the Fc glycan with FcγRs [11] glycan-glycan interactions have recently been shown to modulate the affinity of IgG1 Fc portions to the FcγR, and in particular to FcγRIIIa, with the lack of core-fucose on the IgG1 Asn297 N-glycan promoting high-affinity interaction with the Asn162 N-glycan of the receptor [10, 12]. The high-affinity interaction of afucosylated IgG1 with FcγRIIIa has been shown to form the molecular basis of the enhanced efficacy of afucosylated therapeutic antibodies in killing cancer cells [13, 14], and design of the glycosylation during product development may represent an attractive way of increasing efficacy in new therapeutic IgGs [15, 16]. The reduced fucosylation of IgG1 may also be important in pathological situations, *e.g.* during pregnancies complicated with the formation of maternal IgG against fetal platelets, which we found to be highly skewed towards the afucosylated kind [17].

Recently, Fc sialylation of IgG has received increased attention, as it has been reported that increased sialylation makes IgGs anti-inflammatory agents [18, 19]. In murine models it has been shown that sialylated IgGs bind to DC-SIGN receptors of immune cells and leads to the upregulation of inhibitory FcγRIIb on macrophages [19–21].

Human serum IgG glycosylation is known to change with various physiological and pathological conditions. Both galactosylation and sialylation show a pronounced age and sex dependence with a higher galactosylation and sialylation of IgG in females than in males at young age, and a decrease in galactosylation and sialylation for both sexes with increasing age [22, 23]. In addition, various autoimmune and infectious diseases have been shown to result in decreased IgG galactosylation [24–26]. In contrast, pregnancy is known to be associated with an increase in galactosylation and sialylation of IgG Fc N-glycans, with a concomitant decrease in the incidence of bisecting GlcNAc [27–29]. These glycosylation changes may be typed as anti-inflammatory [18], and one may speculate that these adaptations contribute to suppressing alloimmune reactions during pregnancy [30].

Human IgG is actively transported across the placenta *via* FcRn into the circulation of the fetus, and this IgG provided by the mother is considered to contribute to the immunological protection of the fetus and newborn during the first months after birth [31]. The infant starts producing its own IgG in the first weeks after birth [32], but IgGs produced by the infant are still found at low levels until 8 months of age, when only IgG1 and sometimes IgG3, but not IgG2 and IgG4 can reach similar levels found for adults [33].

Two studies in 1995 [34] and 1996 [35] compared the IgG glycosylation of maternal and fetal IgG. The studies analyzed total glycosylation of IgG and described a lower level of agalactosylated structures [34, 35] and higher percentages of galactosylated N-glycan structures [35] for fetal as compared to maternal IgG. These data indicated that there might be a preferential transport of galactosylated IgG to the fetus. However, these studies analyzed total IgG glycosylation, thus including both Fc glycans and glycans of the IgG variable parts, found in approximately 30 % of all immunoglobulins [36–38]. If the reported increase was due to Fc galactosylation with a possible concomitant increase in sialylation, it may be expected to influence the effector functions of fetal IgG. We, therefore, decided to study the specific glycosylation features of fetal IgG in more detail, focusing only on the Fc glycosylation. These results would also give us insight into whether there are other receptors, besides FcRn, involved in placental transport favouring transport of certain Fc glycoforms. To this end, we chose to analyse only the IgG Fc glycosylation of paired fetal and maternal samples in a site-specific and subclass-specific manner. For this purpose, IgG was purified from plasma by protein G affinity chromatography followed by tryptic cleavage. Fc N-glycopeptides were analyzed by mass spectrometry resulting in glycosylation profiles of IgG1 and IgG4 as well as combined Fc glycosylation profiles of IgG2 and 3 [29]. The analysis of all sample pairs revealed very similar levels of galactosylation, sialylation, fucosylation, and bisecting GlcNAc for IgG between fetus and mother.

## Materials and methods

### Patient samples and IgG isotype analysis

The pairs of plasma samples from mothers and umbilical cord of the new-born were collected right after delivery (average gestation time 37.8 weeks, range 36–39 weeks). All women had an uncomplicated pregnancy and neonatal outcomes for all children were optimal. Signed informed consent was obtained from all women, and the collection of blood samples and clinical data recieved approval by the Ethics Committee of the Leiden University Medical Center (P02-200).

The analysis of total IgG and IgG isotypes were performed using the Siemens nephelometer BNII.

### Purification of IgG

IgGs were affinity captured from total human plasma as described previously [39]. Protein G Sepharose™ Fast Flow beads (GE Healthcare, Uppsala, Sweden) were washed three times with 10 volumes of PBS. 15 μL of beads in 150 μL PBS were incubated with 2 μL of serum in a 96-well filter plate (Multiscreen Solvinert, 0.45 μm pore-size low-binding hydrophilic PTFE; Millipore, Billerica, MA) on a shaker for 1 h. Beads were thoroughly washed 4 times with 200 μL of PBS and then 3 times with 200 μL of water under vacuum (pressure reduction to approximately 900 mbar). IgG was eluted into a 96-well V-bottom plate using 100 μL formic acid (100 mM). Samples were dried by vacuum centrifugation.

### IgG digestion with trypsin

IgGs were digested with trypsin as described previously [8]. A 20 μg aliquot of trypsin (sequencing grade; Promega, Leiden, The Netherlands) was dissolved in 4 mL of 25 mM ammonium bicarbonate. Within 1 min after preparation, 40 μL of this mixture was added per well to the dried purified antibodies. Samples were shaken (1 min), incubated overnight at 37 °C, and stored at −20 °C until usage.

### Fast nano-reverse phase-LC-ESI-MS

Nano-reverse phase-LC-ESI-MS was performed as described previously [29]. Briefly, analysis was achieved on a Ultimate 3000 HPLC system (Thermo Fisher, Waltham, MA), equipped with a Acclaim PepMap100 C18 (5 mm × 300 μm i.d.; Thermo Fisher) solid phase extraction (SPE) trap column and Ascentis Express C18 nano-LC column (50 mm × 75 μm i.d., 2.7 μm HALO fused core particles; Supelco, Bellefonte, USA). Samples were centrifuged at 4,000 rpm for 5 min and aliquots of 500 nL were applied to the trap column for 1 min at 25 μl/min. Separation was achieved with the following gradient of mobile phase A (0.1 % trifluoroacetic acid; Fluka, Steinheim, Germany) and mobile phase B (95 % acetonitrile; Biosolve BV, Valkenswaard, the Netherlands): 0 min 3 % B, 2 min 5 % B, 5 min 20 % B, 6 min 30 % B, 8 min 30 % B, 9 min 0 % B, and 14 min 0 % B. After 8 min the SPE was switched off-line and washed by three full loop injections containing 5 μL 5 % isopropanol (IPA) + 0.1 % formic acid (FA) and 5 μL 50 % IPA + 0.1 % FA. The HPLC was interfaced to a quadrupole-TOF mass spectrometer (micrOTOF-Q; Bruker Daltonics, Bremen, Germany) with a standard ESI source (Bruker Daltonics) and a sheath-flow ESI sprayer (capillary electrophoresis ESI-MS sprayer; Agilent Technologies, Santa Clara, USA) applying the UV outlet tubing (20 μm i.d., 360 μm o.d.) as sprayer needle. A sheath-flow of 50 % IPA, 20 % propionic acid and 30 % water was applied at 2 μL/min to support ESI spray formation and reduce TFA ion suppression. To improve mobile phase evaporation a nitrogen stream was applied as dry gas at 4 L/min with a nebulizer pressure of 0.4 bar. Scan spectra were recorded from 300 to 2,000 Da with 2 average scans at a frequency of 1 Hz. Quadrupole ion energy and collision energy of the MS were set at 2 and 4 eV, respectively. The total analysis time per sample was 16 min.

### Data processing

Data processing was performed as described previously [29]. Briefly, LC-MS datasets were calibrated internally using a list of known glycopeptides and were exported to the open mzXML format by Bruker DataAnalysis 4.0. Each dataset was then aligned to a master dataset of a typical sample (containing many of the (glyco-)peptide species shared between multiple samples) using msalign2 [40]. Glycopeptide species which were pre-defined as peak maxima in specific mass and retention time windows, were extracted from each dataset using the in-house developed software “Xtractor2D”. The software and ancillary scripts are freely available at www.ms-utils.org/Xtractor2D. The complete sample-data matrix was finally evaluated using Microsoft Excel.

Structural assignment of the detected glycoforms was performed on the basis of literature knowledge of IgG N-glycosylation [6–8, 23, 41, 42]. Relative intensities of the glycopeptide species (Table 1) derived from IgG1 (20 glycoforms), IgG4 (10 glycoforms), and IgG2 (20 glycoforms) were obtained by integrating and summing three isotopic peaks of the triple protonated as well as the double protonated species followed by normalization to the total IgG subclass specific glycopeptide intensities.

**Table 1:** Theoretical *m/z* values of human IgG Fc glycopeptides detected by nano-LC-ESI-MS

Glycan species	IgG1 P01857^b^		IgG2/3 P01859^b^/P01860, VAR_003892^b^		IgG4 P01861^b^	
	[M + 2H]^2+^	[M + 3H]^3+^	[M + 2H]^2+^	[M + 3H]^3+^	[M + 2H]^2+^	[M + 3H]^3+^
G0F^c^	1317.527	878.687	1301.532	868.024	1309.529	873.356^a1^
G1F	1398.553	932.705	1382.558	922.042	1390.556	927.373^a2^
G2F	1479.580	986.722	1463.585	976.059	1471.582	981.391
G0FN	1419.067	946.380	1403.072	935.717	1411.069	941.049^a3^
G1FN	1500.093	1000.398	1484.098	989.735	1492.096	995.066^a4^
G2FN	1581.119	1054.416	1565.125	1043.752	1573.122	1049.084
G1FS	1544.101	1029.737	1528.106	1019.073	1536.104	1024.405^a5^
G2FS	1625.127	1083.754	1609.133	1073.091	1617.130	1078.423
G1FNS	1645.641	1097.430	1629.646	1086.767	1637.643	1092.098
G2FNS	1726.667	1151.447	1710.672	1140.784	1718.670	1146.116
G0	1244.498	830.001	1228.503	819.338	n.d.	n.d.
G1	1325.524	884.019	1309.529	873.356^a1^	n.d.	n.d.
G2	1406.551	938.036	1390.556	927.373^a2^	n.d.	n.d.
G0N	1346.038	897.694	1330.043	887.031	n.d.	n.d.
G1N	1427.064	951.712	1411.069	941.049^a3^	n.d.	n.d.
G2N	1508.090	1005.730	1492.096	995.066^a4^	n.d.	n.d.
G1S	1471.072	981.051	1455.077	970.387	n.d.	n.d.
G2S	1552.098	1035.068	1536.104	1024.405^a5^	n.d.	n.d.
G1NS	1572.612	1048.744	1556.617	1038.081	n.d.	n.d.
G2NS	1653.638	1102.761	1637.643	1092.098	n.d.	n.d.

^a1–a5^ isomeric glycopeptide species of IgG4 and IgG2

^b^ SwissProt entry number

^c^ glycan structural features are given in terms of number of galactoses (G0, G1, G2), fucose (F), bisecting *N*-acetylglucosamine (N), and *N*-acetylneuraminic acid, sialic acid (S)

*n.d.* not detected

In addition, the levels of 4 major glycoforms of IgG1, IgG2/3 and IgG4 glycopeptides with one missed cleavage site were monitored as triple and quadruple charged species (Table 2) in order to judge the efficacy of the tryptic digest.

**Table 2:** Theoretical *m/z* values of human IgG Fc glycopeptides with 1 missed cleavage site

Glycan species	IgG1 P01857^a^		IgG2/3 P01859^a^/P01860, VAR_003892^a^		IgG4 P01861^a^	
	[M + 3H]^3+^	[M + 4H]^4+^	[M + 3H]^3+^	[M + 4H]^4+^	[M + 3H]^3+^	[M + 4H]^4+^
G0F^b^	1039.453	779.842	1028.789	771.844	1034.121	775.843
G1F	1093.470	820.355	1082.807	812.357	1088.139	816.356
G2F	1147.488	860.868	1136.825	852.870	1142.156	856.869
G2FS	1244.520	933.642	1233.856	925.644	1239.188	929.643

^a^ SwissProt entry number; the peptide moieties of IgG1, IgG2/3 and IgG4 are TKPREEQYNSTYR ([M + H]^+^ 1671.809), TKPREEQFNSTFR ([M + H]^+^ 1639.819), TKPREEQFNSTYR ([M + H]^+^ 1655.814), respectively

^b^ Glycan structural features are given in terms of number of galactoses (G0, G1, G2), fucose (F), and *N*-acetylneuraminic acid, sialic acid (S)

On the basis of the normalized intensities of IgG Fc glycopeptides the level of galactosylation, sialylation, bisecting *N*-acetylglucosamine, and fucosylation were calculated according to the following formulas: Galactosylation = (G1F + G1FN + G1FS + G1FNS + G1 + G1N + G1S) * 0.5 + G2F + G2FN + G2FS + G2FNS + G2 + G2N + G2S. Agalactosylated structures = G0F + G0FN + G0 + G0N. Digalactosylated structures = G2F + G2FN + G2FS + G2FNS + G2 + G2N + G2S. Sialylation = G1FS + G2FS + G1FNS + G2FNS + G1S + G2S. Bisecting GlcNAc = G0FN + G1FN + G2FN + G1FNS + G2FNS + G0N + G1N + G2N. Fucosylation = G0F + G1F + G2F + G0FN + G1FN + G2FN + G1FS + G2FS. The non-fucosylated species of IgG4 remained below the limit of detection and were, therefore, not included in the IgG4 calculations.

In addition, we calculated from the isotype-specific IgG G0 levels the overall IgG G0 levels for both fetus and mother, in order to facilitate the comparison of our results with those obtained by others [34, 35]. Calculations were performed according to the following formula: Overall IgG G0 = (IgG1 G0 + IgG1 G0F + IgG1 G0FN + IgG1 G0N) × relative abundance IgG1 + (IgG2/3 G0 + IgG2/3 G0F + IgG2/3 G0FN + IgG2/3 G0N) × (relative abundance IgG2/3) + (IgG4 G0F + IgG4 G0FN) × relative abundance IgG4.

## Results and discussion

**Fig. 1:**
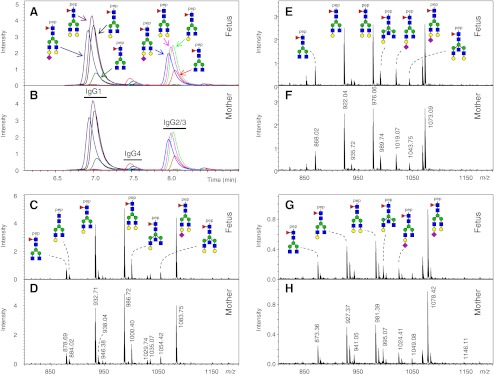
Nano-LC-ESI-MS of tryptic digests of IgG obtained from fetal and maternal blood. Extracted ion chromatograms of the triple- and double-protonated glycopeptide species G0F, G1F, G2F and G2FS of IgG1, IgG2/3, and IgG4 are displayed for fetus (**a**) and mother (**b**). Integration ranges for the MS signals are indicated by horizontal bars. The corresponding mass spectra showing the triple protonated IgG1 (**c**, **d**), IgG2/3 (**e**, **f**), and IgG4 (**g**, **h**) glycopeptide species are shown for fetus (**c**, **e**, **g**) and mother (**d**, **f**, **h**). *Blue square*, *N-*acetylglucosamine; *red triangle*, fucose; *green circle*, mannose; *yellow circle*, galactose; *purple diamond*, *N*-acetylneuraminic acid.

IgG was purified from 20 plasma samples of maternal and umbilical vein blood (fetus) using Protein G Sepharose. IgG was subjected to tryptic cleavage, and resulting IgG Fc glycopeptides were analyzed using a recently established nano-LC-MS method employing a sheath-flow ESI sprayer [29]. IgG1 Fc glycopeptides were found to elute at approximately 7 min, IgG4 Fc glycopeptides at 7.5 min, and IgG2/3 Fc glycopeptides at 8 min (Fig. 1a, b). IgG2 and IgG3 tryptic Fc glycopeptides have the identical peptide moieties [8] and, therefore, these IgG isotypes were registered together. Glycan structures were assigned on the basis of literature knowledge of IgG glycan structures [6, 23, 41, 42] and the established elution orders of IgG Fc glycopeptides in reverse phase-LC-MS [7, 8].

All 20 samples were checked for the completeness of the tryptic digest by monitoring the IgG1 and IgG2 Fc glycopeptides with one missed tryptic cleavage site, according to Stadlmann *et al.* [7] (Table 2). Miscleaved glycopeptides were found in only a minority of samples. When observed, the signal intensities of miss-cleaved glycopeptides were found to be at least 200 times lower than those of the fully cleaved glycopeptides, and the signal-noise ratio was low throughout. Due to their low abundance the signal of miss-cleaved glycopeptides were not included in the quantitative analysis.

Fetal and maternal IgG showed very similar chromatographic profiles as evidenced by extracted ion chromatograms of the major IgG1, IgG2/3, and IgG4 Fc glycopeptides (Fig. 1a, b). For all the 10 fetal and 10 maternal IgG1 samples Fc glycosylation profiles were obtained (see Fig. 1c and d for an example). Signals obtained for the triple protonated IgG1 Fc glycopeptides were observed in the range of *m/z* 800 to 1,200 (Fig. 1c, d), whilst the signals of double protonated species were registered in the range of *m/z* 1,200 to 1,800 (see Table 1) [29]. The double and triple charged signals were integrated and summed for all the 20 registered IgG1 Fc glycopeptide species. The sum of all IgG1 Fc glycopeptides species was set to 100 %, and the degree of galactosylation, sialylation, bisecting *N*-acetylglucosamine, and fucosylation were determined. Similarly, 20 IgG2/3 Fc glycopeptides and 10 IgG4 Fc glycopeptides were analyzed (Fig. 1e-h). While IgG2/3 Fc glycopeptides were analyzed for all 10 fetal and maternal IgG pairs, only seven of the fetal and maternal IgG4 Fc glycopeptide clusters showed sufficient intensity for glycopeptide analysis.

The level of galactosylation reflects the percentage of antennae, which are decorated with a galactose residue. Therefore, monogalactosylated and digalactosylated glycans were weighed differently to reflect their different degree of galactosylation. While the percentage of digalactosylated structures was fully included in the galactosylation term, monogalactosylated structures were only weighed half, due to the fact that they carry a galactosylated as well as a non-galactosylated antenna (see Materials and methods for the equation). Maternal IgG1 showed average levels of galactosylation of 73.8 %. This value represents elevated levels of IgG galactosylation as they have been described for advanced pregnancies. The determined galactosylation level is very much in line with the average IgG1 Fc galactosylation levels of 70.7 % recently analyzed for 26 pregnant women during the third trimester [29]. Overall, the tendency is that IgG galactosylation peaks at delivery and normalizes a few months after. IgG galactosylation levels at 3 as well as 6 months after delivery were found to be 61.2 % and 61.9 %, respectively, representing the non-pregnant levels of IgG galactosylation. Similarly, the IgG2/3 galactosylation levels of 66.4 % observed in this study are in line with previously determined values of 63.2 % for the third pregnancy trimester, and are significantly higher than the values of 52.1 % observed 6 months after delivery (non-pregnant status). For IgG4, the average galactosylation levels observed here (68.4 %) are significantly higher than those observed before for the third trimester of pregnancy (54.7 %) and 6 months after delivery (46.4 %) [29]. One may assume that this difference is linked to the slightly different sampling time points (third trimester *versus* at delivery). The possible physiological role of such an increased IgG4 Fc galactosylation at the end of the pregnancy is unclear.

**Fig. 2:**
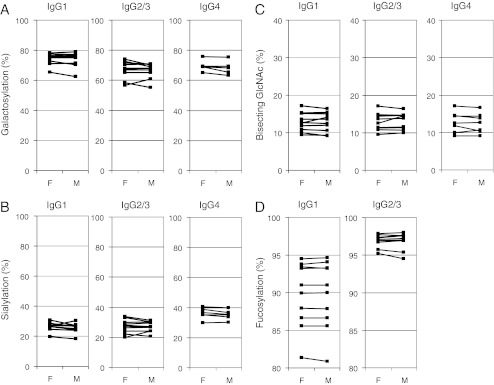
Fc glycosylation analysis of IgG from paired cord blood (fetus, F) and maternal blood (M). Galactosylation (**a**), sialylation (**b**), bisecting GlcNAc (**c**), and fucosylation (**d**) were compared for tryptic Fc glycopeptides of IgG1, IgG2/3 and IgG4

The mean levels of Fc galactosylation of fetal and maternal IgG1 were found to be 74.6 % *versus* 73.8 % (Table 3; Fig. 2a). Likewise, IgG2/3 of fetus and mother showed very similar levels of galactosylation (average for the 10 analyzed pairs of 67.1 % and 66.4 %, respectively). For the seven pairs of fetal and maternal IgG4 average galactosylation values were found to be 69.5 % and 68.4 %, respectively. These results indicate no significant differences in Fc galactosylation between fetal and maternal IgG for the major isotypes (IgG1 and IgG2/3; Table 3).

**Table 3:** Comparison of the Fc glycosylation features of fetal and maternal IgG. Mean values ± standard deviation are given. The standard error of the mean is given in parentheses

Glycosylation feature	Subclass	Mean relative abundance ± standard deviation (%)			*t*-test
		Fetus	Mother	Fetus–Mother	
Galactosylation	IgG1 Fc	74.6 ± 3.9	73.8 ± 4.7	0.8 ± 1.3 (0.4)	*p* = 0.10
	IgG2/3 Fc	67.1 ± 5.8	66.4 ± 5.0	0.7 ± 2.4 (0.8)	*p* = 0.38
	IgG4 Fc	69.5 ± 3.2	68.4 ± 3.7	1.1 ± 1.2 (0.4)	*p* = 0.04
Sialylation	IgG1 Fc	26.4 ± 2.9	25.4 ± 3.1	1.0 ± 2.0 (0.6)	*p* = 0.14
	IgG2/3 Fc	27.4 ± 4.4	27.0 ± 3.4	0.4 ± 2.0 (0.6)	*p* = 0.59
	IgG4 Fc	36.7 ± 3.7	35.9 ± 3.4	0.7 ± 0.9 (0.3)	*p* = 0.06
Bisecting GlcNAc	IgG1 Fc	12.9 ± 2.5	12.8 ± 2.6	0.1 ± 0.7 (0.2)	*p* = 0.72
	IgG2/3 Fc	13.0 ± 2.3	13.2 ± 2.2	−0.2 ± 0.7 (0.2)	*p* = 0.33
	IgG4 Fc	12.7 ± 2.8	12.5 ± 2.6	0.2 ± 0.7 (0.3)	*p* = 0.38
Fucosylation	IgG1 Fc	89.7 ± 4.3	89.7 ± 4.5	0.0 ± 0.2 (0.1)	*p* = 0.90
	IgG2/3 Fc	96.9 ± 0.8	96.9 ± 1.1	−0.0 ± 0.3 (0.1)	*p* = 0.84

In order to facilitate the comparison of our results with those of Williams *et al.* [34] and Kibe *et al.* [35], we calculated the relative abundances of agalactosylated as well as digalactosylated structures. For the IgG1, IgG2/3 and IgG4 subclasses, we found that the levels of agalactosylated structures were very similar for fetal and maternal samples. There was a tendency of slightly lower levels of galactosylation of fetal IgG, but the mean differences in agalactosylated species between fetal and maternal IgG1, IgG2/3 and IgG4 were only 0.1 %, 0.4 %, and 0.8 %, respectively (Table 4). In contrast, Williams *et al.* [34] described significantly lower levels of agalactosylated structures for IgG of the fetus (mean value of 13.1 %) as compared to maternal IgG (mean value of 16.7 %; difference of 3.6 % is statistically highly significant; *p* = 0.000012; see Table 4). Likewise, Kibe *et al.* [35] analyzed 26 sets of paired samples revealing lower levels of agalactosylated structures for fetal as compared to maternal IgG (10 % *versus* 12 %; Table 4).

**Table 4:** Comparison of the results on fetal and maternal IgG glycosylation from this study and two previous studies. Mean values ± standard deviation are given. The standard error of the mean is given in parentheses

Sample pairs	Fetus	Mother	Fetus–Mother	*t*-test	Source
Agalactosylated structures (%)					
*n* = 10	IgG 13.1 ± 5.6	16.7 ± 5.9	−3.6 ± 1.3	*p* = 0.000012	Williams *et al.* 1995 [34]
*n* = 26	IgG 10.0 ± 2	12 ± 2	−2.0	*p* = 0.0015	Kibe *et al.* 1996 [35]
*n* = 10	IgG1 Fc 8.0 ± 2.4	8.4 ± 3.1	−0.4 ± 1.1 (0.36)	*p* = 0.27	this study
*n* = 10	IgG2/3 Fc 14.6 ± 4.4	14.7 ± 3.5	−0.1 ± 2.8 (0.87)	*p* = 0.87	this study
*n* = 7	IgG4 Fc 14.4 ± 2.3	15.2 ± 2.6	−0.8 ± 0.8 (0.31)	*p* = 0.04	this study
Digalactosylated structures (%)					
*n* = 26	IgG 61 ± 4	58 ± 4	3.0	*p* = 0.007	Kibe *et al.* 1996 [35]
*n* = 10	IgG1 Fc 57.3 ± 5.5	56.3 ± 6.3	1.0 ± 1.5 (0.46)	*p* = 0.05	this study
*n* = 10	IgG2/3 Fc 49.4 ± 7.3	48.2 ± 6.7	1.2 ± 2.3 (0.74)	*p* = 0.14	this study
*n* = 7	IgG4 Fc 53.4 ± 4.5	52.0 ± 5.1	1.4 ± 1.5 (0.57)	*p* = 0.05	this study

Comparison of the levels of digalactosylated structures also revealed some differences with literature: Kibe *et al.* [35] found mean levels of digalactosylated structures to be 3 % higher on fetal as compared to maternal IgG (*p* = 0.007). In our study, mean differences were much lower, or around 1 %, none reaching the level of significance.

However, the conceptual, as well as methodological differences between the two previous studies and our current study should be noted. In the previous studies, the N-linked glycans were released from IgG by hydrazinolysis [34] and PNGase A treatment [35]. Glycans were radioactively labeled by Williams *et al.* [34], and the levels of agalactosylated structures were assessed by gel permeation chromatography after simplifying the oligosaccharide mixture by employing a cocktail of exoglycosidases (α-sialidase, α-fucosidase and β-*N*-acetylhexosaminidase). Kibe *et al.* [35] analyzed the oligosaccharides by reverse phase HPLC profiling after fluorescent labelling with 2-aminopyridine and enzymatic desialylation. In both cases, total N-glycans were registered, *i.e.*, both Fc glycosylation, and Fab glycosylation. As our study indicates that Fc glycosylation of all the IgG subclasses is remarkably similar in fetal and maternal IgG, one may speculate about the cause of the differences observed in earlier studies. First, the changes in overall IgG glycosylation profiles as observed by Williams *et al.* and Kibe *et al.* may in part have been caused by differences in subclass ratios [34, 35]. This is a possibility because the levels of Fc galactosylation tend to be higher for IgG1, than for IgG2/3 and IgG4 (Table 3). In addition, the analysis of the IgG isotype distribution indicated that the portion of IgG1 is elevated in cord blood (72.3 %) as compared to maternal blood (64.8 %; paired *t*-test *p* = 0.0000015; Table 5). On the other hand, the relative IgG2 abundances are lower for the fetus than for the mother (22.0 % *versus* 28.4 %; paired *t*-test *p* = 0.0000065), and the same holds true for IgG3 (2.7 % *versus* 3.8 %; paired *t*-test *p* = 0.0022). In the present study, glycosylation analysis was performed in a subclass-specific manner, and therefore not influenced by the ratio of subclasses. To compare our results with the previous studies [34, 35], we used the information on the relative isotype distribution and calculated the overall levels of IgG Fc agalactosylated structures by taking the isotype ratios into account (see Materials and Methods for details). The average overall levels of IgG Fc agalactosylated species were calculated to be 9.7 % for the fetus *versus* 10.6 % for the mother. Hence, for the specific set of 10 paired mother/child IgG samples analyzed in this study, the observed Fc glycosylation profiles, together with the changes in relative abundances of IgG subclasses, result in a 0.9 % decrease of overall IgG Fc agalactosylated structures. Therefore, the difference in subclass ratios seems to only partly explain the pronounced differences in overall galactosylation reported previously [34, 35].

**Table 5:** Concentrations of total IgG and IgG isotypes of fetus and mother. Concentrations are given in mg/ml. Percentages are given in parentheses

Mother/child couple	Fetus					Mother^a^				
	IgG1	IgG2	IgG3	IgG4	Total	IgG1	IgG2	IgG3	IgG4	Total
1	3.86 (54.8)	2.44 (34.6)	0.15 (2.1)	0.60 (8.5)	7.05	1.97 (46.2)	1.78 (41.8)	0.12 (2.8)	0.39 (9.2)	4.26
2	6.09 (75.8)	1.69 (21.0)	0.21 (2.6)	0.04 (0.5)	8.02	5.99 (68.5)	2.43 (27.8)	0.31 (3.5)	0.01 (0.1)	8.72
3	3.36 (73.5)	1.00 (21.9)	0.20 (4.4)	0.01 (0.2)	4.57	2.60 (66.2)	1.06 (27.0)	0.26 (6.6)	0.01 (0.3)	3.92
4	3.62 (68.8)	1.39 (26.4)	0.13 (2.5)	0.12 (2.3)	5.25	2.82 (58.8)	1.71 (35.6)	0.15 (3.1)	0.12 (2.5)	4.79
5	5.02 (60.5)	2.48 (29.9)	0.33 (4.0)	0.47 (5.7)	8.30	2.33 (49.3)	1.90 (40.2)	0.22 (4.7)	0.28 (5.9)	4.73
6	6.01 (81.0)	1.24 (16.7)	0.16 (2.2)	0.01 (0.1)	7.41	3.93 (73.0)	1.30 (24.2)	0.14 (2.6)	0.01 (0.2)	5.37
7	8.26 (82.8)	1.13 (11.3)	0.15 (1.5)	0.44 (4.4)	9.98	5.63 (79.0)	1.07 (15.0)	0.13 (1.8)	0.30 (4.2)	7.11
8	8.40 (75.5)	2.40 (21.6)	0.18 (1.6)	0.14 (1.3)	11.12	4.20 (70.0)	1.60 (26.7)	0.12 (2.0)	0.08 (1.3)	6.00
9	6.40 (76.4)	1.60 (19.1)	0.26 (3.1)	0.12 (1.4)	8.38	2.80 (68.6)	1.00 (24.5)	0.22 (5.4)	0.06 (1.5)	4.08
10	4.70 (74.0)	1.08 (17.0)	0.22 (3.5)	0.35 (5.5)	6.35	5.20 (68.1)	1.60 (20.9)	0.42 (5.5)	0.42 (5.5)	7.64
Average	5.57 (72.3)	1.65 (22.0)	0.20 (2.7)	0.23 (3.0)	7.65	3.75 (64.8)	1.55 (28.4)	0.21 (3.8)	0.17 (3.1)	5.67

^a^ Reference standard values IgG1: 4.9–11.4; IgG2: 1.50–6.4; IgG3: 0.20–1.10; IgG4: 0.080–1.40

The IgG isotype concentrations of our samples are rather low compared to normal adult standard values (Table 5), which is in line with the known decrease to 60–70 % for both IgA (not transported to the fetus) and IgG in pregnant women at term [43]. Notably the average IgG concentrations found by us (7.65 mg/ml fetal IgG and 5.67 mg/ml maternal IgG) were significantly lower than those found by Kibe *et al.* [35] (13.15 mg/ml fetal IgG and 10.70 mg/ml maternal IgG), the reason for this discrepancy being unknown.

Lastly, differences in Fab glycosylation between fetal and maternal IgG may explain why previous studies found the markedly increased levels of overall galactosylation of fetal IgG. If true, this would indicate either a preferential transport of Fab-galactosylated IgG or retention of a Fab-agalactosylated IgG, possibly by an unknown receptor either actively involved in, or interfering with, IgG transport. This needs to be investigated in more detail. It has to be noted, however, that robust and straight-forward methods with reasonable throughput for the specific Fab glycosylation analysis of polyclonal human IgG are still lacking and, therefore, such analyses are scarce and performed on only very small numbers of samples [38, 44].

Next to IgG Fc galactosylation, the levels of sialylation of IgG1, IgG2/3 and IgG4 were assessed in our study. Similar to the observations for IgG Fc galactosylation, we found no significant changes in the levels of Fc sialylation for fetal as compared to maternal IgG (Table 3). Interestingly, sialylation levels were found to be higher for maternal IgG4 (35.9 %) as compared to IgG1 (25.4 %) and IgG2/3 (27.0 %) (Table 3). The subclass with the highest levels of galactosylation (IgG1) is not the one with the highest levels of sialylation, indicating a differential regulation of galactosylation and sialylation between IgG subclasses. Notably, in our previous study sialylation levels of IgG4 were found to be 26.4 % in the third pregnancy trimester (on average 9 weeks before delivery), whilst the values of IgG4 sialylation were down to 21.1 %, 19.9 % and 20.1 % at 6 weeks, 3 months, and 6 months after delivery, respectively [29]. Hence, the high levels of IgG4 sialylation found in the present study may point to a transient increase in IgG4 sialylation with delivery.

The comparison of the levels of bisecting GlcNAc revealed mean levels between 12.5 % and 13.2 % for fetal and maternal IgG of all analyzed subclasses (Table 3). No differences in the level of bisecting GlcNAc were found between fetal and maternal IgG (Fig. 2; Table 3).

Finally, a comparison of the levels of fucosylation was performed for both IgG1 and IgG2/3. No differences in core-fucosylation were detected between fetal and maternal IgG. IgG1 fucosylation levels were found to be in the range of 80 % to 95 %, while IgG2/3 fucosylation levels were between 94 % and 98 % for all the analyzed paired samples of fetus and mother. Interestingly, no correlation was observed between IgG1 and IgG2/3 fucosylation levels (not shown), indicating that Fc fucosylation in IgG-secreting B cells is likely to be regulated largely independently between subclasses – perhaps as a result of differential stimulation. This might be explained by the preferential class switching to IgG2 induced by T-helper independent antigens, compared to T-cell dependent antibody responses where IgG1 dominates [45].

We therefore conclude that trans-placental transport of human IgG does not favor certain Fc glycoforms. This is in line with reports demonstrating that FcRn, the receptor proposed to be solely responsible for placental transport and *in vivo* half life, rescues glycosylated and aglycosylated forms of IgG equally well [46]. This also fits with the structural requirements for FcRn binding to IgG, which does not involve the Fc glycans [9]. These results also exclude a significant role for FcγR isoforms that do display discriminatory binding activities to different glycoforms [10, 12, 18].

## Conclusion

Previous studies found a higher degree of N-glycan galactosylation of total fetal IgG as compared to maternal IgG. In contrast, when analyzing IgG Fc glycosylation in a subclass-specific manner, we did not detect skewing of glycosylation profiles in general, also not for the level of galactosylation levels. This indicates that the materno-fetal IgG transport is not Fc-glycosylation-selective in healthy pregnancies. These results lend support to the commonly held belief that FcRn is the only contributing receptor to the placental transport of antibodies to the fetus. However, results of previous studies indicate that alternative mechanisms may be in place for Fab-glycosylated IgG.
